# Crystal structure of idelalisib *tert*-butanol monosolvate dihydrate

**DOI:** 10.1107/S2056989019002743

**Published:** 2019-02-28

**Authors:** Sven Nerdinger, Marijan Stefinovic, Frank Richter, Jacek Olma, Michal Brysz, Tracy Walker, Volker Kahlenberg, Thomas Gelbrich

**Affiliations:** a Sandoz GmbH, Biochemiestrasse 10, 6250 Kundl, Austria; b Selvita S.A., Park Life Science, Bobrzynskiego 14, 30-348 Kraków, Poland; c Almac Group, Almac House, 20 Seagoe Industrial Estate, Craigavon BT63 5QD, United Kingdom; d University of Innsbruck, Institute of Mineralogy and Petrography, Innrain 52, 6020 Innsbruck, Austria; e University of Innsbruck, Institute of Pharmacy, Innrain 52, 6020 Innsbruck, Austria

**Keywords:** crystal structure, hydrogen bonding, hydrate, solvate, pharmaceuticals

## Abstract

In the title structure, 5-fluoro-3-phenyl-2-[(1*S*)-1-(9*H*-purin-6-yl­amino)­prop­yl]quinazolin-4(3*H*)-one (= idelalisib) *tert*-butanol monosolvate dihydrate, C_22_H_18_FN_7_O·C_4_H_10_O·2H_2_O, the idelalisib mol­ecule displays planar quinazoline and purine systems which are nearly perpendicular to one another. Seven distinct hydrogen-bonding inter­actions link the idelalisib, *t*-BuOH and water mol­ecules into a complex chain structure with the topology of a 2,3,4,5-connected 4-nodal net having the point symbol (3.4.5^2^.6^2^)(3.4.5^2^.6^4^.7^2^)(3.5.6)(5).

## Abstract 2

Mol­ecules of the three components, idelalisib, *tert*-butanol and water, are linked into a hydrogen-bonded chain structure with the topology of a 2,3,4,5-connected 4-nodal net.

## Chemical context   

Idelalisib is a novel, orally available small-mol­ecule inhibitor of phosphatidylinositol 3-kinase delta (PI3Kdelta). This compound was developed for the oral treatment of chronic lymphocytic leukemia and is currently marketed under the trade name Zydelig by Gilead Sciences, Inc. Carra *et al.* (2013 ▸) reported the existence of seven solid forms of idelalisib and unit-cell parameters for five of these, namely for two polymorphs, an *i*-PrOH solvate hydrate, a DMF and a DMSO solvate. The current study is part of an investigation of a modified synthetic route for idelalisib, which ultimately resulted in improved yields compared to the original synthesis by Kesicki & Zhichkin (2005 ▸).
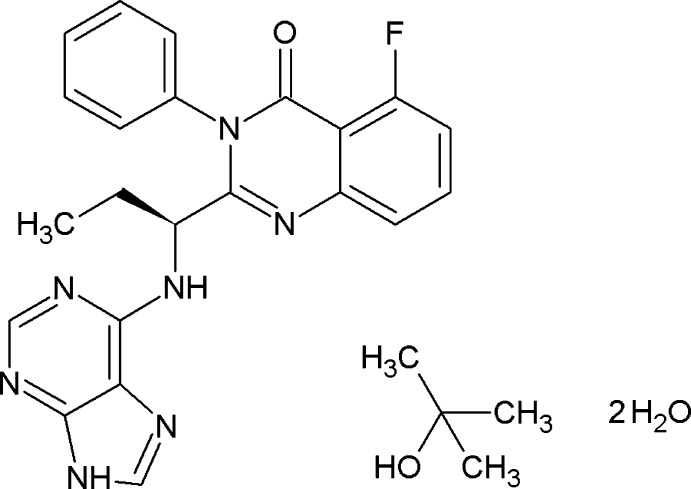


## Structural commentary   

**Figure 1:**
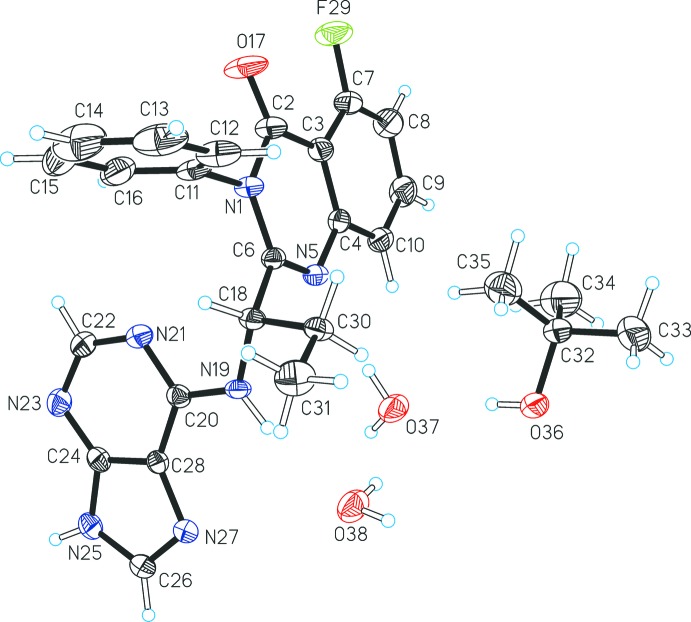
Asymmetric unit of (I) with displacement ellipsoids drawn at the 50% probability level and hydrogen atoms as spheres of arbitrary size.

The asymmetric unit of the title compound, (I), contains one formula unit, *i.e.* a mol­ecule each of idelalisib and of *t*-BuOH as well as two water mol­ecules, denoted as *w*1 (O37) and *w*2 (O38) (Fig. 1 ▸). The conformation of the idelalisib mol­ecule can be described in terms of the relative orientations adopted by the three planar fragments of the quinazoline group N1>C10, the phenyl ring C11>C16, and the purine group C20 >C28. The mean planes of the phenyl and purine units both lie approximately perpendicular to the quinazoline mean plane and form dihedral angles of 88.10 (8) and 86.97 (6)°, respectively, with the latter. The dihedral angle between the phenyl and purine mean planes is 73.75 (7)°. The torsion angles around the C30—C18 bond are C31—C30—C18—C6 = 165.5 (2)° (propyl group) and C31—C30—C18—N19 = −71.6 (3)°.

## Supra­molecular features   

**Figure 2:**
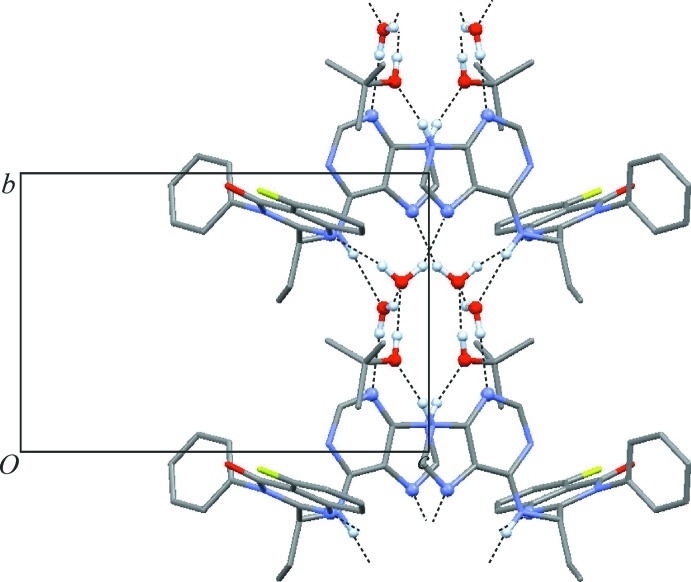
Hydrogen-bonded chain structure of (I), viewed along the *a* axis. H, N and O atoms directly engaged in hydrogen bonding are drawn as spheres. All other H atoms are omitted for clarity.

**Figure 3:**
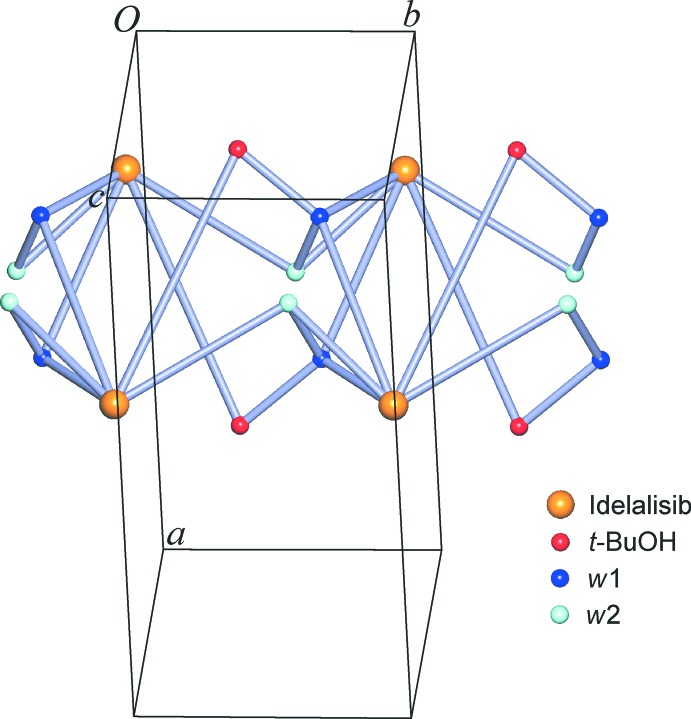
2,3,4,5-Connected 4-nodal topological net representing the hydrogen-bonded chain structure of (I) which is based on the seven inter­molecular inter­actions listed in Table 1 ▸.

The endocyclic NH group of the purine unit donates a hydrogen bond to the *t*-BuOH mol­ecule, *via* N25—H25⋯O36(−*x* + 1, *y* + 1, −*z* + 2). Additionally, the secondary amino function attached to the pyrimidine ring of the purine fragment donates a hydrogen bond to a *w*2 water mol­ecule, *via* N19—H19⋯O38. In turn, the idelalisib mol­ecule accepts three hydrogen bonds. Its quinazoline group is linked to the *w*1 water mol­ecule *via* an O37—H37*A*⋯N5 bond, and additionally each of N23 and N27 of the purine group is hydrogen-bonded to a water mol­ecule of type *w*2 [O38—H38*A*⋯N23(*x*, *y* − 1, *z*)] or *w*1 [O37—H37*B*⋯N27(−*x* + 1, *y*, −*z* + 2)]. Moreover, the water mol­ecule *w*1 is an acceptor for two H-bonds, O36—H36⋯O37 from a *t*-BuOH mol­ecule and O38—H38*B*⋯O37 from a *w*2-type water mol­ecule. There are no hydrogen bonds between neighbouring idelalisib mol­ecules. Overall, the seven classical hydrogen-bonding inter­actions listed in Table 1 ▸ result in a chain that possesses a central twofold rotational axis and propagates parallel to the *b* axis (Fig. 2 ▸). Each idelalisib mol­ecule represents a five-connected node within this hydrogen-bonded chain structure and is linked to one *t*-BuOH, two *w*1 and two *w*2 mol­ecules. The *t*-BuOH mol­ecule is a two-connected node and serves as a bridge between an idelalisib and a *w*1 mol­ecule. The water mol­ecule *w*1 is four-connected (2 × idelalisib, 1 × *t*-BuOH, 1 × *w*2), whilst *w*2 serves as a three-connected node (2 × idelalisib, 1 × *w*1). The hydrogen-bonded chain of (I) has the topology of the 2,3,4,5-connected 4-nodal 1D net depicted in Fig. 3 ▸, which has the point symbol (3.4.5^2^.6^2^)(3.4.5^2^.6^4^.7^2^)(3.5.6)(5). The topology of the hydrogen-bonded structure was determined and classified with the programs *ADS* and *IsoTest* of the *TOPOS* package (Blatov, 2006 ▸) in the manner described by Baburin & Blatov (2007 ▸).

## Database survey   

The most recent version 5.40 (November 2018) of the Cambridge Structural Database (Groom *et al.*, 2016 ▸) does not contain any data for solid forms of idelalisib.

The bond parameters of the quinazoline system are in agreement with the relevant features in two polymorphs of 3-phenyl­quinazolin-4(3*H*)-one (Zhou *et al.*, 2008 ▸; Yu *et al.*, 2018 ▸), in 2-[2-(4-nitro­phen­yl)vin­yl]-3-phenyl­quinazolin-4(3*H*)-one (Nosova *et al.*, 2012 ▸) and 2-di­ethyl­amino-3-phenyl­quinazolin-4(3*H*)-one (Xie & Li, 2006 ▸). Likewise, the structural parameters of the purine skeleton are consistent with the relevant reference structures such as 1- and 7-(β-d-ribo­furanos­yl)adenine (Framski *et al.*, 2006 ▸).

## Synthesis and crystallization   

**Figure 4:**
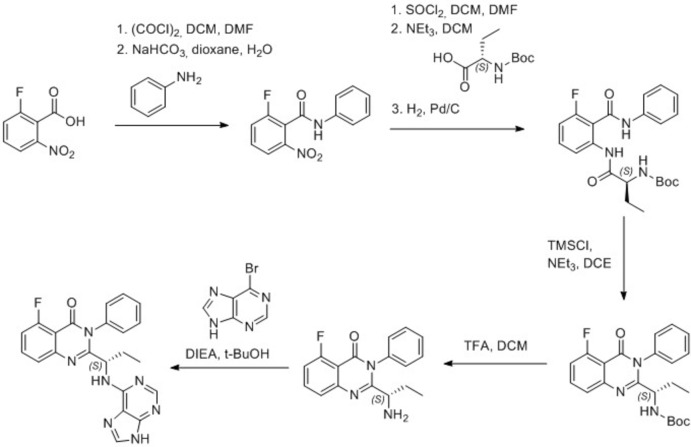
Synthetic scheme for the preparation of idelalisib.

The preparation of idelalisib was carried out according to the scheme displayed in Fig. 4 ▸, which represents a modification of the original synthesis by Kesicki & Zhichkin (2005 ▸), and yielded the polymorphic form I described by Carra *et al.* (2013 ▸). To amorphous idelalisib (180 mg), which was obtained by lyophilization of form I in dioxane, were added 500 µL of *t*-BuOH/water 95:5 (*v*/*v*) at 296 K. The amorphous material was dissolved. Precipitation of solid material was observed after 5 min of stirring of the solution. The suspension was then stirred at 296 K for five days, which was followed by centrifugation and separation of the precipitate. Subsequent drying of the solid material yielded the title compound (I) as a crystalline, free-flowing white powder (120 mg, 55%).

## Refinement   

Crystal data, data collection and structure refinement details are summarized in Table 2 ▸. All hydrogen atoms were identified in Fourier-difference maps. Methyl H atoms were idealized (C—H = 0.98 Å) and included as rigid groups allowed to rotate but not to tip and were refined with *U*_iso_(H) = 1.5*U*_eq_(C) of the parent carbon atom. All other hydrogen atoms bonded to carbon atoms were positioned geometrically (C—H = 0.95 Å) and refined with *U*_iso_(H) = 1.5*U*_eq_(C) of the parent carbon atom. Hydrogen atoms of OH and NH groups were refined with restrained distances [O—H = 0.84 (1) Å; N—H = 0.88 (1) Å] and their *U*_iso_ parameters were refined freely. The absolute structure was established by anomalous-dispersion effects (Table 2 ▸).

The largest residual peak of 0.73 e Å^−3^ is located 1.00 Å from C30. An alternative refinement of a disorder model with a split C30 position was attempted but resulted in a few unreasonably short intra­mol­ecular H⋯H distances for the minor disorder fragment. This feature could not be eliminated even with the application of an anti-bumping restraint.

## Supplementary Material

Crystal structure: contains datablock(s) I. DOI: 10.1107/S2056989019002743/wm5487sup1.cif

Structure factors: contains datablock(s) I. DOI: 10.1107/S2056989019002743/wm5487Isup2.hkl

Supporting information file. DOI: 10.1107/S2056989019002743/wm5487Isup3.cml

CCDC reference: 1898812

Additional supporting information:  crystallographic information; 3D view; checkCIF report

## Figures and Tables

**Table 1:** Hydrogen-bond geometry (Å, °)

*D*—H⋯*A*	*D*—H	H⋯*A*	*D*⋯*A*	*D*—H⋯*A*
N19—H19⋯O38	0.88 (2)	2.09 (2)	2.963 (3)	170 (3)
N25—H25⋯O36^i^	0.87 (2)	1.88 (2)	2.750 (3)	173 (4)
O36—H36⋯O37	0.82 (3)	1.92 (3)	2.741 (3)	174 (5)
O37—H37*A*⋯N5	0.86 (2)	2.09 (2)	2.939 (3)	172 (3)
O37—H37*B*⋯N27^ii^	0.90 (2)	2.06 (3)	2.888 (3)	153 (4)
O38—H38*A*⋯N23^iii^	0.87 (2)	2.04 (2)	2.905 (3)	172 (3)
O38—H38*B*⋯O37	0.88 (2)	2.09 (3)	2.921 (4)	158 (4)

Symmetry codes: (i) 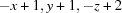; (ii) 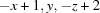; (iii) 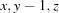.

**Table 2:** Experimental details

Crystal data	
Chemical formula	C_22_H_18_FN_7_O·C_4_H_10_O·2H_2_O
*M*_r_	525.58
Crystal system, space group	Monoclinic, *C*2
Temperature (K)	173
*a*, *b*, *c* (Å)	21.3758 (6), 9.2781 (3), 13.9722 (5)
β (°)	102.654 (3)
*V* (Å^3^)	2703.75 (15)
*Z*	4
Radiation type	Mo *K*α
μ (mm^−1^)	0.09
Crystal size (mm)	0.34 × 0.26 × 0.18

Data collection	
Diffractometer	Rigaku Oxford Diffraction Xcalibur, Ruby, Gemini ultra
Absorption correction	Multi-scan (*CrysAlis PRO*; Rigaku OD, 2015 ▸)
*T*_min_, *T*_max_	0.835, 1.000
No. of measured, independent and observed [*I* > 2σ(*I*)] reflections	8990, 5111, 4751
*R*_int_	0.020
(sin θ/λ)_max_ (Å^−1^)	0.617

Refinement	
*R*[*F* ^2^ > 2σ(*F* ^2^)], *wR*(*F* ^2^), *S*	0.039, 0.098, 1.07
No. of reflections	5111
No. of parameters	375
No. of restraints	10
H-atom treatment	H atoms treated by a mixture of independent and constrained refinement
Δρ_max_, Δρ_min_ (e Å^−3^)	0.27, −0.18
Absolute structure	Flack *x* determined using 1997 quotients [(*I* ^+^)−(*I* ^−^)]/[(*I* ^+^)+(*I* ^−^)] (Parsons *et al.*, 2013 ▸)
Absolute structure parameter	−0.1 (4)

Computer programs: *CrysAlis PRO* (Rigaku OD, 2015 ▸), *SIR2002* (Burla *et al.*, 2003 ▸), *SHELXL2014* (Sheldrick, 2015 ▸), *XP* (Bruker, 1998 ▸), *Mercury* (Macrae *et al.*, 2006 ▸), *TOPOS* (Blatov, 2006 ▸), *PLATON* (Spek, 2009 ▸) and *publCIF* (Westrip, 2010 ▸).

## supplementary crystallographic information

### Crystal data

**Table:**

C_22_H_18_FN_7_O·C_4_H_10_O·2H_2_O	*F*(000) = 1112
*M**_r_* = 525.58	*D*_x_ = 1.291 Mg m^−^^3^
Monoclinic, *C*2	Mo *K*α radiation, λ = 0.71073 Å
*a* = 21.3758 (6) Å	Cell parameters from 5110 reflections
*b* = 9.2781 (3) Å	θ = 2.7–28.3°
*c* = 13.9722 (5) Å	µ = 0.09 mm^−^^1^
β = 102.654 (3)°	*T* = 173 K
*V* = 2703.75 (15) Å^3^	Irregular fragment, colourless
*Z* = 4	0.34 × 0.26 × 0.18 mm

### Data collection

**Table:**

Rigaku Oxford Diffraction Xcalibur, Ruby, Gemini ultra diffractometer	5111 independent reflections
Radiation source: fine-focus sealed X-ray tube, Enhance (Mo) X-ray Source	4751 reflections with *I* > 2σ(*I*)
Graphite monochromator	*R*_int_ = 0.020
Detector resolution: 10.3575 pixels mm^-1^	θ_max_ = 26.0°, θ_min_ = 2.2°
ω scans	*h* = −20→26
Absorption correction: multi-scan (*CrysAlis PRO*; Rigaku OD, 2015)	*k* = −10→11
*T*_min_ = 0.835, *T*_max_ = 1.000	*l* = −16→12
8990 measured reflections	

### Refinement

**Table:**

Refinement on *F*^2^	Secondary atom site location: difference Fourier map
Least-squares matrix: full	Hydrogen site location: mixed
*R*[*F*^2^ > 2σ(*F*^2^)] = 0.039	H atoms treated by a mixture of independent and constrained refinement
*wR*(*F*^2^) = 0.098	*w* = 1/[σ^2^(*F*_o_^2^) + (0.0448*P*)^2^ + 1.4882*P*] where *P* = (*F*_o_^2^ + 2*F*_c_^2^)/3
*S* = 1.07	(Δ/σ)_max_ < 0.001
5111 reflections	Δρ_max_ = 0.27 e Å^−^^3^
375 parameters	Δρ_min_ = −0.18 e Å^−^^3^
10 restraints	Absolute structure: Flack *x* determined using 1997 quotients [(*I*^+^)-(*I*^-^)]/[(*I*^+^)+(*I*^-^)] (Parsons *et al.*, 2013)
Primary atom site location: structure-invariant direct methods	Absolute structure parameter: −0.1 (4)

### Special details

**Table:**

Geometry. All esds (except the esd in the dihedral angle between two l.s. planes) are estimated using the full covariance matrix. The cell esds are taken into account individually in the estimation of esds in distances, angles and torsion angles; correlations between esds in cell parameters are only used when they are defined by crystal symmetry. An approximate (isotropic) treatment of cell esds is used for estimating esds involving l.s. planes.

### Fractional atomic coordinates and isotropic or equivalent isotropic displacement parameters (Å^2^)

**Table:**

	*x*	*y*	*z*	*U*_iso_*/*U*_eq_	
N1	0.31764 (11)	0.8629 (3)	0.59496 (16)	0.0225 (5)	
C2	0.25203 (13)	0.8986 (3)	0.5816 (2)	0.0269 (6)	
C3	0.22506 (13)	0.8657 (3)	0.6664 (2)	0.0238 (6)	
C4	0.26486 (13)	0.8111 (3)	0.7519 (2)	0.0220 (6)	
N5	0.32934 (10)	0.7807 (2)	0.75732 (16)	0.0205 (5)	
C6	0.35268 (13)	0.8049 (3)	0.68133 (18)	0.0190 (5)	
C7	0.16088 (14)	0.8885 (3)	0.6679 (2)	0.0301 (7)	
C8	0.13634 (14)	0.8634 (4)	0.7481 (3)	0.0357 (7)	
H8	0.0922	0.8796	0.7461	0.043*	
C9	0.17702 (16)	0.8135 (4)	0.8331 (2)	0.0350 (7)	
H9	0.1609	0.7983	0.8905	0.042*	
C10	0.24025 (14)	0.7861 (3)	0.8347 (2)	0.0266 (6)	
H10	0.2674	0.7498	0.8928	0.032*	
C11	0.34784 (13)	0.8965 (4)	0.5149 (2)	0.0281 (7)	
C12	0.35269 (15)	0.7897 (4)	0.4473 (2)	0.0367 (8)	
H12	0.3343	0.6973	0.4514	0.044*	
C13	0.38504 (18)	0.8210 (5)	0.3736 (3)	0.0519 (11)	
H13	0.3891	0.7492	0.3269	0.062*	
C14	0.4111 (2)	0.9557 (6)	0.3681 (3)	0.0635 (14)	
H14	0.4335	0.9760	0.3179	0.076*	
C15	0.40512 (18)	1.0609 (5)	0.4347 (3)	0.0559 (12)	
H15	0.4229	1.1538	0.4297	0.067*	
C16	0.37325 (15)	1.0323 (4)	0.5091 (2)	0.0395 (8)	
H16	0.3690	1.1048	0.5553	0.047*	
O17	0.22461 (10)	0.9541 (3)	0.50590 (16)	0.0443 (6)	
C18	0.42053 (13)	0.7557 (3)	0.68211 (19)	0.0206 (6)	
H18	0.4364	0.8091	0.6300	0.025*	
N19	0.46407 (11)	0.7818 (3)	0.77607 (17)	0.0210 (5)	
H19	0.4813 (14)	0.709 (3)	0.813 (2)	0.021 (8)*	
C20	0.48111 (12)	0.9170 (3)	0.8058 (2)	0.0200 (6)	
N21	0.45009 (11)	1.0276 (3)	0.75290 (17)	0.0249 (5)	
C22	0.46761 (14)	1.1613 (3)	0.7819 (2)	0.0275 (7)	
H22	0.4447	1.2358	0.7424	0.033*	
N23	0.51255 (12)	1.2060 (3)	0.85799 (19)	0.0278 (6)	
C24	0.54192 (13)	1.0929 (3)	0.9097 (2)	0.0216 (6)	
N25	0.58955 (11)	1.0964 (3)	0.99228 (18)	0.0242 (5)	
H25	0.6096 (17)	1.174 (3)	1.017 (3)	0.043 (11)*	
C26	0.60377 (13)	0.9565 (3)	1.0169 (2)	0.0251 (6)	
H26	0.6358	0.9289	1.0724	0.030*	
N27	0.56911 (11)	0.8628 (3)	0.95710 (17)	0.0227 (5)	
C28	0.52953 (12)	0.9492 (3)	0.88899 (19)	0.0193 (6)	
F29	0.12046 (8)	0.9365 (2)	0.58583 (14)	0.0450 (5)	
C30	0.41885 (13)	0.5942 (3)	0.6584 (2)	0.0233 (6)	
H30A	0.4119	0.5392	0.7159	0.028*	
H30B	0.3824	0.5742	0.6028	0.028*	
C31	0.48062 (15)	0.5434 (4)	0.6322 (2)	0.0379 (8)	
H31A	0.5164	0.5557	0.6889	0.057*	
H31B	0.4887	0.6004	0.5771	0.057*	
H31C	0.4766	0.4414	0.6136	0.057*	
C32	0.28332 (15)	0.3199 (3)	0.8427 (2)	0.0338 (7)	
C33	0.2706 (2)	0.1621 (5)	0.8256 (4)	0.0719 (15)	
H33A	0.2641	0.1171	0.8862	0.108*	
H33B	0.3073	0.1170	0.8060	0.108*	
H33C	0.2320	0.1490	0.7736	0.108*	
C34	0.23156 (19)	0.3891 (5)	0.8861 (3)	0.0569 (11)	
H34A	0.2445	0.4875	0.9073	0.085*	
H34B	0.2254	0.3326	0.9426	0.085*	
H34C	0.1913	0.3920	0.8365	0.085*	
C35	0.28984 (18)	0.3982 (5)	0.7512 (3)	0.0461 (9)	
H35A	0.3226	0.3509	0.7230	0.069*	
H35B	0.3024	0.4983	0.7674	0.069*	
H35C	0.2487	0.3964	0.7035	0.069*	
O36	0.34320 (12)	0.3280 (2)	0.91451 (19)	0.0419 (6)	
H36	0.353 (2)	0.414 (3)	0.920 (3)	0.063 (14)*	
O37	0.38596 (12)	0.6073 (2)	0.93073 (17)	0.0386 (6)	
H37A	0.3696 (17)	0.665 (4)	0.884 (2)	0.044 (11)*	
H37B	0.402 (2)	0.665 (4)	0.982 (2)	0.068 (14)*	
O38	0.50757 (12)	0.5153 (2)	0.88771 (17)	0.0361 (5)	
H38A	0.5126 (16)	0.424 (3)	0.877 (3)	0.040 (10)*	
H38B	0.4728 (15)	0.523 (4)	0.911 (3)	0.064 (14)*	

### Atomic displacement parameters (Å^2^)

**Table:**

	*U*^11^	*U*^22^	*U*^33^	*U*^12^	*U*^13^	*U*^23^
N1	0.0200 (11)	0.0277 (12)	0.0181 (11)	0.0050 (10)	0.0007 (9)	0.0021 (9)
C2	0.0222 (13)	0.0359 (17)	0.0212 (14)	0.0069 (13)	0.0014 (11)	0.0031 (12)
C3	0.0207 (13)	0.0223 (14)	0.0277 (15)	0.0022 (12)	0.0036 (11)	0.0008 (12)
C4	0.0228 (13)	0.0172 (13)	0.0246 (14)	−0.0006 (11)	0.0023 (11)	0.0004 (11)
N5	0.0192 (11)	0.0211 (12)	0.0193 (12)	0.0023 (9)	0.0005 (9)	0.0003 (9)
C6	0.0203 (13)	0.0168 (12)	0.0179 (13)	0.0009 (11)	0.0000 (10)	−0.0006 (10)
C7	0.0206 (14)	0.0323 (18)	0.0351 (17)	0.0040 (13)	0.0009 (12)	0.0059 (13)
C8	0.0213 (14)	0.0426 (18)	0.0464 (19)	0.0062 (14)	0.0142 (14)	0.0094 (15)
C9	0.0323 (16)	0.0390 (18)	0.0376 (18)	−0.0005 (14)	0.0160 (14)	0.0070 (15)
C10	0.0253 (15)	0.0266 (16)	0.0278 (15)	0.0023 (12)	0.0051 (12)	0.0046 (12)
C11	0.0218 (13)	0.0403 (18)	0.0208 (14)	0.0120 (13)	0.0015 (11)	0.0085 (12)
C12	0.0312 (16)	0.055 (2)	0.0221 (15)	0.0162 (16)	0.0016 (13)	0.0000 (15)
C13	0.041 (2)	0.088 (3)	0.0276 (18)	0.026 (2)	0.0085 (16)	0.0061 (19)
C14	0.041 (2)	0.116 (4)	0.039 (2)	0.022 (3)	0.0210 (17)	0.028 (3)
C15	0.043 (2)	0.073 (3)	0.052 (2)	0.002 (2)	0.0115 (18)	0.035 (2)
C16	0.0358 (17)	0.048 (2)	0.0333 (18)	0.0057 (16)	0.0041 (14)	0.0154 (16)
O17	0.0270 (11)	0.0747 (18)	0.0298 (12)	0.0214 (12)	0.0027 (9)	0.0201 (12)
C18	0.0182 (13)	0.0242 (14)	0.0175 (13)	0.0002 (11)	0.0001 (10)	0.0001 (11)
N19	0.0188 (11)	0.0201 (13)	0.0201 (12)	0.0018 (10)	−0.0045 (9)	0.0010 (9)
C20	0.0168 (12)	0.0222 (14)	0.0211 (13)	0.0005 (11)	0.0047 (10)	0.0001 (11)
N21	0.0238 (12)	0.0241 (13)	0.0250 (12)	0.0030 (11)	0.0016 (10)	0.0035 (10)
C22	0.0270 (15)	0.0233 (16)	0.0313 (16)	0.0024 (13)	0.0043 (13)	0.0059 (12)
N23	0.0283 (13)	0.0207 (13)	0.0342 (14)	−0.0003 (11)	0.0067 (11)	0.0022 (10)
C24	0.0189 (13)	0.0249 (14)	0.0217 (14)	−0.0024 (12)	0.0063 (11)	−0.0023 (12)
N25	0.0234 (12)	0.0220 (12)	0.0269 (13)	−0.0043 (11)	0.0048 (10)	−0.0050 (10)
C26	0.0212 (14)	0.0269 (16)	0.0250 (15)	−0.0026 (12)	0.0001 (11)	−0.0005 (12)
N27	0.0195 (11)	0.0233 (12)	0.0242 (12)	−0.0005 (10)	0.0024 (9)	0.0010 (9)
C28	0.0163 (12)	0.0218 (15)	0.0207 (13)	−0.0009 (11)	0.0056 (10)	0.0020 (11)
F29	0.0225 (9)	0.0661 (14)	0.0441 (11)	0.0116 (9)	0.0020 (8)	0.0223 (10)
C30	0.0204 (13)	0.0233 (14)	0.0233 (14)	0.0023 (12)	−0.0014 (11)	−0.0037 (12)
C31	0.0369 (17)	0.039 (2)	0.0394 (19)	0.0112 (15)	0.0117 (14)	−0.0049 (15)
C32	0.0291 (16)	0.0276 (17)	0.0388 (17)	0.0007 (13)	−0.0054 (13)	0.0049 (13)
C33	0.057 (3)	0.035 (2)	0.104 (4)	−0.009 (2)	−0.026 (3)	0.004 (2)
C34	0.050 (2)	0.071 (3)	0.051 (2)	0.003 (2)	0.0133 (18)	0.008 (2)
C35	0.0412 (19)	0.056 (2)	0.0387 (19)	−0.0058 (18)	0.0037 (15)	0.0013 (17)
O36	0.0383 (13)	0.0236 (13)	0.0525 (15)	−0.0013 (10)	−0.0151 (11)	0.0061 (11)
O37	0.0517 (15)	0.0267 (12)	0.0284 (12)	−0.0038 (11)	−0.0106 (11)	0.0012 (10)
O38	0.0453 (14)	0.0233 (12)	0.0370 (13)	0.0031 (11)	0.0032 (11)	0.0010 (10)

### Geometric parameters (Å, º)

**Table:**

N1—C6	1.382 (3)	N21—C22	1.333 (4)
N1—C2	1.413 (3)	C22—N23	1.334 (4)
N1—C11	1.443 (4)	C22—H22	0.9500
C2—O17	1.207 (3)	N23—C24	1.348 (4)
C2—C3	1.459 (4)	C24—N25	1.362 (4)
C3—C7	1.393 (4)	C24—C28	1.378 (4)
C3—C4	1.401 (4)	N25—C26	1.360 (4)
C4—C10	1.392 (4)	N25—H25	0.87 (2)
C4—N5	1.392 (4)	C26—N27	1.316 (4)
N5—C6	1.288 (4)	C26—H26	0.9500
C6—C18	1.518 (4)	N27—C28	1.382 (4)
C7—F29	1.351 (3)	C30—C31	1.520 (4)
C7—C8	1.358 (4)	C30—H30A	0.9900
C8—C9	1.388 (5)	C30—H30B	0.9900
C8—H8	0.9500	C31—H31A	0.9800
C9—C10	1.371 (4)	C31—H31B	0.9800
C9—H9	0.9500	C31—H31C	0.9800
C10—H10	0.9500	C32—O36	1.445 (4)
C11—C16	1.381 (5)	C32—C33	1.498 (5)
C11—C12	1.388 (5)	C32—C35	1.504 (5)
C12—C13	1.392 (5)	C32—C34	1.516 (5)
C12—H12	0.9500	C33—H33A	0.9800
C13—C14	1.378 (7)	C33—H33B	0.9800
C13—H13	0.9500	C33—H33C	0.9800
C14—C15	1.374 (7)	C34—H34A	0.9800
C14—H14	0.9500	C34—H34B	0.9800
C15—C16	1.389 (5)	C34—H34C	0.9800
C15—H15	0.9500	C35—H35A	0.9800
C16—H16	0.9500	C35—H35B	0.9800
C18—N19	1.454 (3)	C35—H35C	0.9800
C18—C30	1.534 (4)	O36—H36	0.82 (3)
C18—H18	1.0000	O37—H37A	0.86 (2)
N19—C20	1.346 (4)	O37—H37B	0.90 (2)
N19—H19	0.88 (2)	O38—H38A	0.87 (2)
C20—N21	1.351 (4)	O38—H38B	0.88 (2)
C20—C28	1.409 (4)		
			
C6—N1—C2	122.6 (2)	C22—N21—C20	118.0 (2)
C6—N1—C11	120.8 (2)	N21—C22—N23	129.6 (3)
C2—N1—C11	116.5 (2)	N21—C22—H22	115.2
O17—C2—N1	119.6 (3)	N23—C22—H22	115.2
O17—C2—C3	126.8 (3)	C22—N23—C24	110.8 (2)
N1—C2—C3	113.6 (2)	N23—C24—N25	127.6 (3)
C7—C3—C4	117.3 (3)	N23—C24—C28	126.5 (3)
C7—C3—C2	123.1 (3)	N25—C24—C28	105.9 (2)
C4—C3—C2	119.6 (2)	C26—N25—C24	106.0 (2)
C10—C4—N5	118.1 (2)	C26—N25—H25	129 (2)
C10—C4—C3	120.0 (3)	C24—N25—H25	125 (2)
N5—C4—C3	121.9 (2)	N27—C26—N25	113.9 (3)
C6—N5—C4	118.5 (2)	N27—C26—H26	123.0
N5—C6—N1	123.6 (2)	N25—C26—H26	123.0
N5—C6—C18	118.9 (2)	C26—N27—C28	103.2 (2)
N1—C6—C18	117.2 (2)	C24—C28—N27	110.9 (2)
F29—C7—C8	117.8 (3)	C24—C28—C20	116.8 (2)
F29—C7—C3	119.1 (3)	N27—C28—C20	132.3 (3)
C8—C7—C3	123.1 (3)	C31—C30—C18	112.0 (3)
C7—C8—C9	118.7 (3)	C31—C30—H30A	109.2
C7—C8—H8	120.7	C18—C30—H30A	109.2
C9—C8—H8	120.7	C31—C30—H30B	109.2
C10—C9—C8	120.5 (3)	C18—C30—H30B	109.2
C10—C9—H9	119.8	H30A—C30—H30B	107.9
C8—C9—H9	119.8	C30—C31—H31A	109.5
C9—C10—C4	120.4 (3)	C30—C31—H31B	109.5
C9—C10—H10	119.8	H31A—C31—H31B	109.5
C4—C10—H10	119.8	C30—C31—H31C	109.5
C16—C11—C12	121.5 (3)	H31A—C31—H31C	109.5
C16—C11—N1	119.4 (3)	H31B—C31—H31C	109.5
C12—C11—N1	119.1 (3)	O36—C32—C33	105.3 (3)
C11—C12—C13	118.6 (4)	O36—C32—C35	109.4 (3)
C11—C12—H12	120.7	C33—C32—C35	112.6 (4)
C13—C12—H12	120.7	O36—C32—C34	108.3 (3)
C14—C13—C12	120.2 (4)	C33—C32—C34	110.8 (4)
C14—C13—H13	119.9	C35—C32—C34	110.3 (3)
C12—C13—H13	119.9	C32—C33—H33A	109.5
C15—C14—C13	120.5 (4)	C32—C33—H33B	109.5
C15—C14—H14	119.7	H33A—C33—H33B	109.5
C13—C14—H14	119.7	C32—C33—H33C	109.5
C14—C15—C16	120.4 (4)	H33A—C33—H33C	109.5
C14—C15—H15	119.8	H33B—C33—H33C	109.5
C16—C15—H15	119.8	C32—C34—H34A	109.5
C11—C16—C15	118.8 (4)	C32—C34—H34B	109.5
C11—C16—H16	120.6	H34A—C34—H34B	109.5
C15—C16—H16	120.6	C32—C34—H34C	109.5
N19—C18—C6	112.2 (2)	H34A—C34—H34C	109.5
N19—C18—C30	109.7 (2)	H34B—C34—H34C	109.5
C6—C18—C30	108.4 (2)	C32—C35—H35A	109.5
N19—C18—H18	108.8	C32—C35—H35B	109.5
C6—C18—H18	108.8	H35A—C35—H35B	109.5
C30—C18—H18	108.8	C32—C35—H35C	109.5
C20—N19—C18	120.7 (2)	H35A—C35—H35C	109.5
C20—N19—H19	119 (2)	H35B—C35—H35C	109.5
C18—N19—H19	120 (2)	C32—O36—H36	107 (3)
N19—C20—N21	118.2 (2)	H37A—O37—H37B	105 (3)
N19—C20—C28	123.5 (2)	H38A—O38—H38B	107 (3)
N21—C20—C28	118.3 (2)		
			
C6—N1—C2—O17	177.1 (3)	C11—C12—C13—C14	−0.4 (5)
C11—N1—C2—O17	0.0 (4)	C12—C13—C14—C15	−0.5 (6)
C6—N1—C2—C3	−1.2 (4)	C13—C14—C15—C16	0.8 (6)
C11—N1—C2—C3	−178.3 (3)	C12—C11—C16—C15	−1.0 (5)
O17—C2—C3—C7	3.7 (5)	N1—C11—C16—C15	176.7 (3)
N1—C2—C3—C7	−178.2 (3)	C14—C15—C16—C11	0.0 (5)
O17—C2—C3—C4	−175.0 (3)	N5—C6—C18—N19	−41.0 (3)
N1—C2—C3—C4	3.1 (4)	N1—C6—C18—N19	144.2 (2)
C7—C3—C4—C10	−1.9 (4)	N5—C6—C18—C30	80.3 (3)
C2—C3—C4—C10	176.8 (3)	N1—C6—C18—C30	−94.4 (3)
C7—C3—C4—N5	178.2 (3)	C6—C18—N19—C20	−69.6 (3)
C2—C3—C4—N5	−3.0 (4)	C30—C18—N19—C20	169.8 (2)
C10—C4—N5—C6	−179.3 (3)	C18—N19—C20—N21	9.1 (4)
C3—C4—N5—C6	0.5 (4)	C18—N19—C20—C28	−171.0 (2)
C4—N5—C6—N1	1.6 (4)	N19—C20—N21—C22	−179.2 (3)
C4—N5—C6—C18	−172.7 (2)	C28—C20—N21—C22	0.8 (4)
C2—N1—C6—N5	−1.3 (4)	C20—N21—C22—N23	−0.3 (5)
C11—N1—C6—N5	175.7 (3)	N21—C22—N23—C24	−0.5 (4)
C2—N1—C6—C18	173.2 (2)	C22—N23—C24—N25	−179.3 (3)
C11—N1—C6—C18	−9.8 (4)	C22—N23—C24—C28	0.8 (4)
C4—C3—C7—F29	−177.8 (3)	N23—C24—N25—C26	−179.6 (3)
C2—C3—C7—F29	3.4 (5)	C28—C24—N25—C26	0.3 (3)
C4—C3—C7—C8	1.5 (5)	C24—N25—C26—N27	−0.1 (3)
C2—C3—C7—C8	−177.2 (3)	N25—C26—N27—C28	−0.2 (3)
F29—C7—C8—C9	179.8 (3)	N23—C24—C28—N27	179.5 (3)
C3—C7—C8—C9	0.5 (5)	N25—C24—C28—N27	−0.5 (3)
C7—C8—C9—C10	−2.0 (5)	N23—C24—C28—C20	−0.3 (4)
C8—C9—C10—C4	1.5 (5)	N25—C24—C28—C20	179.7 (2)
N5—C4—C10—C9	−179.7 (3)	C26—N27—C28—C24	0.4 (3)
C3—C4—C10—C9	0.5 (4)	C26—N27—C28—C20	−179.9 (3)
C6—N1—C11—C16	−90.8 (3)	N19—C20—C28—C24	179.5 (3)
C2—N1—C11—C16	86.4 (3)	N21—C20—C28—C24	−0.5 (4)
C6—N1—C11—C12	87.0 (3)	N19—C20—C28—N27	−0.2 (5)
C2—N1—C11—C12	−95.9 (3)	N21—C20—C28—N27	179.7 (3)
C16—C11—C12—C13	1.2 (4)	N19—C18—C30—C31	−71.6 (3)
N1—C11—C12—C13	−176.5 (3)	C6—C18—C30—C31	165.5 (2)

### Hydrogen-bond geometry (Å, º)

**Table:**

*D*—H···*A*	*D*—H	H···*A*	*D*···*A*	*D*—H···*A*
N19—H19···O38	0.88 (2)	2.09 (2)	2.963 (3)	170 (3)
N25—H25···O36^i^	0.87 (2)	1.88 (2)	2.750 (3)	173 (4)
O36—H36···O37	0.82 (3)	1.92 (3)	2.741 (3)	174 (5)
O37—H37*A*···N5	0.86 (2)	2.09 (2)	2.939 (3)	172 (3)
O37—H37*B*···N27^ii^	0.90 (2)	2.06 (3)	2.888 (3)	153 (4)
O38—H38*A*···N23^iii^	0.87 (2)	2.04 (2)	2.905 (3)	172 (3)
O38—H38*B*···O37	0.88 (2)	2.09 (3)	2.921 (4)	158 (4)

Symmetry codes: (i) −*x*+1, *y*+1, −*z*+2; (ii) −*x*+1, *y*, −*z*+2; (iii) *x*, *y*−1, *z*.
